# Changes in Fatty Acids Content in Organic Rosehip (*Rosa* spp.) Seeds during Ripening

**DOI:** 10.3390/plants9121793

**Published:** 2020-12-17

**Authors:** Jurgita Kulaitienė, Brigita Medveckienė, Dovilė Levickienė, Nijolė Vaitkevičienė, Violeta Makarevičienė, Elvyra Jarienė

**Affiliations:** 1 Institute of Agriculture and Food Sciences, Vytautas Magnus University Agriculture Academy, Donelaičio Str. 58, 44248 Kaunas, Lithuania; jurgita.kulaitiene@vdu.lt (J.K.); dovile.levickiene@vdu.lt (D.L.); nijole.vaitkeviciene@vdu.lt (N.V.); elvyra.jariene@vdu.lt (E.J.); 2 Institute of Environment and Ecology, Vytautas Magnus University Agriculture Academy, Donelaičio Str. 58, 44248 Kaunas, Lithuania; violeta.makareviciene@vdu.lt

**Keywords:** rosehip seeds, linoleic acid, oleic acid, palmitic acid, ripening stage

## Abstract

Studies on the determination of the optimal harvest time of rosehips are very limited. Therefore, the aim of this research was to ascertain the effect of the ripening stage on the quality and content of fatty acids of organic rosehip seeds. A two-factor field experiment with two rosehip species and cultivars (*Rosa rugosa*, *Rosa canina*, and *Rosa rugosa* cv. ‘Rubra’, *Rosa rugosa* cv. ‘Alba’) was conducted during two growing seasons (2018–2019) on an organic farm. The fruits were harvested five times per season. The fatty acid composition of rosehip seeds was determined using a Gas Chromatograph with Split/Splitless Injector Liners. The highest amounts of fat were recorded in all rosehip seeds at ripening stage IV. The most dominant fatty acids in the seed samples were polyunsaturated fatty acids (PUFAs) (73.88–79.52%), followed by monounsaturated fatty acids (MUFAs) (14.67–18.89%) and saturated fatty acids (SUFAs) (5.22–7.36%). The highest amount of PUFAs was established in *Rosa rugosa* cv. ‘Alba’ seeds harvested at fully ripe stage V. It can be concluded that the rosehip seeds may be utilized as a source of fatty acids, especially PUFAs.

## 1. Introduction

Lately, there has been a rising interest in the application of novel raw materials oils in the food and cosmetic industries. Oils received from seeds of Rosa plants are common on the market and recommended for nutritional, cosmetic, and pharmaceutical purposes [1,2]. The rosehip seeds may be offered as dietary supplements and as an additive in functional food.

Rosehip fruits are comprised of 30–35% seeds and 65–70% pericarp. The total seed oil of rosehip ranges from 4.97% to 7.95% depending on the species [3]. The lipid fraction of rosehip seeds contains high amounts of polyunsaturated fatty acids (PUFAs). The essential fatty acids of rosehips must be supplied through the diet because humans cannot synthesize them. The essential fatty acids are long-chain PUFAs derived from linoleic, linolenic, and arachidonic acids, which regulate various body functions, such as blood viscosity, blood pressure, and inflammatory and immune responses [4,5,6]. Furthermore, the rosehip seeds oil has a healing power to treat skin problems such as dermatitis, acne, burns, and eczema [7]. Moreover, the seeds of rosehip are used for animal nutrition [8].

Various studies have reported alarming residues of agricultural chemicals in the air, water, soil, agricultural products, and as well as in the human body [9,10]. To create healthier ecosystems, it is necessary to initiate a global transition toward sustainable agriculture. One of the best practices in ensuring environmental sustainability is organic farming because it sustains soil fertility, ecosystems, and human health [11]. Although a few studies have examined the influence of organic agriculture on the fatty acid amount of various edible oils (canola, coconut, mustard seed, olive, and sesame oils), results differ, with some researchers reporting no difference [12], while others notified a higher amount of PUFAs in organic olive oils [13].

By the way, climatic conditions (temperature, rainfall) and soil fertility and type also can affect oil content and fatty acids composition in different seeds [14].

As a result, investigation of the optimal harvesting date is important to obtain rosehip appropriate for the desired health product. Identification of the best harvesting time is crucial for increasing its processed product quality and food value by obtaining the optimal time for fatty acids, vitamin C, sugars, carotenoids, anthocyanins, phenolic, and other compounds. There is a lack of research on the investigation of the optimal harvest time of rosehips [15,16].

Currently, there is a growing interest in healthier and nutritional oil from uncommon raw materials with higher amounts of fatty acids. This research is novel and original due to the fact that fatty acid composition in the seeds of organic rosehip harvested at different ripening stages has not been studied before. Herein, a study was intended to present the fatty acids content and composition properties of rosehip seeds grown in Lithuania, in order to valorize these products as sources of nutrients and nutraceuticals. Therefore, the aim of this research was to determine the effect of the ripening stage on the quality and quantity of fatty acids of seeds of two rosehip species and two cultivars grown under an organic management system.

## 2. Results and Discussion

### 2.1. Total Fat Content

**Figure 1:**
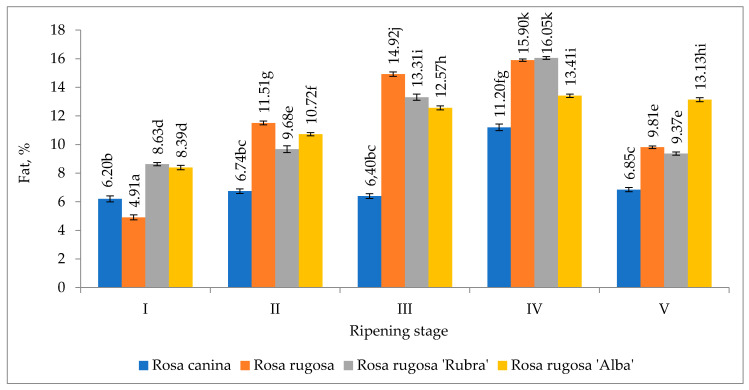
Amount of fat in the organic rosehip seeds. Note: The differences between the means of seed samples and between the means of ripening stages marked by not the same letter are significant at *p* ≤ 0.05.

The two rosehip species and cultivars were grown under the same conditions; however, the effect of the ripening stage on the accumulation of fat in the rosehip seeds was variable (Figure 1). The content of fat in rosehip seeds ranged from 4.91% to 16.05%.

A study conducted by Polish scientists with fruits of eleven wild rose species, harvested in September, found the amount of fat in them to range from 6.50% to 12.90% [17]. In our study, we observed that the content of fat in the rosehip seed samples tended to increase during the growing season till ripening stage IV, and at stage V, it decreased. It could be because, during the final ripening stage of the seeds, some of the triglycerides undergo β-oxidation (degradation), producing acetyl coenzyme A (acetyl-CoA), hence there is a reduction in total oil content [18]. The results indicated that the highest fat content was established significantly in all rosehip seed samples of ripening stage IV. The lowest amount of fat in all rosehip seed samples was determined significantly at the beginning of the experiment.

### 2.2. Unsaturated Fatty Acids

#### 2.2.1. Polyunsaturated Fatty Acids

The studied organic rosehip seeds were found to contain mostly PUFAs (73.88–79.52% of the total fatty acid content) (Table 1), followed by monounsaturated fatty acids (MUFAs) and saturated fatty acids (SFAs). The contents of PUFAs determined in our study are comparable to those reported by other researchers [19,20]. However, the total PUFAs content in the seed samples in our study was higher than the amounts (65.02%) reported by Güney [21]. These differences could be caused by different environmental, soil, and climate conditions, harvest time, genetic factors as well as different oil extraction methods [5,21].

A total of eight PUFAs were found in the studied seeds (Table 1). Among the PUFAs, linoleic acid was recorded as the major fatty acid in the seed samples of various ripening stages as reported by Szentmihalyi et al. [15] and Çelik et al. [3]. In our study, this fatty acid made up about 50% of the total fatty acids in the seeds. The next most abundant fatty acid was linolenic acid in the range from 19.31 (*Rosa canina* seeds at ripening stage V) to 30.64% (*Rosa rugosa* ‘Rubra’ seeds at ripening stage V) of the total fatty acids. In the study conducted by Sharma et al. [22], linoleic acid (45.38–54.58%) and linolenic acid (13.67–24.75%) were the most abundant fatty acids as well. Güneş et al. [19] observed linoleic acid (38.90–57.31%) and linolenic acid (13.65–23.12%) as the most dominant PUFAs in the seeds of different rosehip species.

Our research showed that most PUFAs varied considerably among rosehips samples and ripening stages (Table 1). The fruit stage of ripening impacts directly the quality of the rosehip seeds oil since important chemical variations take place during maturation. Moreover, species/cultivars can react dissimilarly in different environmental conditions, which lead to oils with different characteristics [23]. The seeds of the *Rosa rugosa* ‘Alba’ showed significantly the highest total amount of PUFAs for all ripening stages. The *Rosa rugosa* ‘Alba’ and *Rosa rugosa* ‘Rubra’ seeds had the highest sum of PUFAs at ripening stage V (79.52 and 78.51%, respectively), while the lowest contents were determined at stages I and II (78.92 and 77.44%, respectively). In contrast, the total amounts of PUFAs in *Rosa rugosa* and *Rosa canina* seeds were significantly the lowest at ripening stage V (78.10 and 73.88%, respectively), but they did not significantly differ among ripening stages I, II, III, and IV. Güneş et al. [19] found that during maturation, the total amount of PUFAs in the rosehip seed samples decreased.

The contents of the other PUFAs, including eicosadienoate, eicosatrienoate, arachidonic, eicosapentaenoic, docosadienoate, and docosahexaenoic acids were found to be below 0.200% (Table 1).

The linoleic acid increased with advancing fruit ripening stages (50.63–54.28%) in *Rosa canina* seeds, while the linolenic acid significantly decreased with the maturity (24.31–19.31%). The seeds of the *Rosa rugosa* ‘Alba’ showed the highest amounts of linoleic and linolenic acids at ripening stages III and V, respectively. In *Rosa rugosa* seeds, the amount of linoleic acid was not affected by fruit maturity, while linolenic acid amount significantly decreased with the maturity and it was significantly higher at ripening stage II. In a study carried out by Güneş et al. [18], the amounts of linoleic acid in the analyzed rosehip seeds decreased with advancing maturity, while the content of linolenic acid either increased or did not change.

The amounts of eicosadienoate acid in *Rosa canina* and *Rosa rugosa* ‘Alba’ seeds remained stable during the ripening stages and did not differ significantly. However, the amount of this fatty acid in *Rosa rugosa* and *Rosa rugosa* ‘Rubra’ seeds significantly decreased with advancing ripening from 0.183% to 0.163% and from 0.183% to 0.160%, respectively.

The amounts of eicosatrienoic and arachidonic acids were found to decrease in the *Rosa rugosa* ‘Rubra’ and *Rosa canina* seeds as fruit maturation advanced. However, the amounts of these acids were stable in *Rosa rugosa* ‘Alba’ and *Rosa rugosa* seeds for all ripening stages. Our results showed that the amounts of eicosapentaenoic, docosadienoate, and docosahexaenoic acids depended on the genetic characteristics of rosehip, whereas species/genotype is dependent on genes and enzymes that decrease or increase the synthesis of different fatty acids [24]. The amounts of eicosapentaenoic and docosadienoate acids in the seeds of *Rosa rugosa* ‘Alba’ were the highest and significantly different from the other samples. However, eicosapentaenoic acid was not detected in the seeds of *Rosa rugosa*. The amounts of these three fatty acids also changed significantly during the fruit ripening period. The *Rosa canina* seeds had the highest amounts of eicosapentaenoic acid at ripening stage V (0.020%), while *Rosa rugosa* ‘Alba’ seeds at ripening stage I (0.020%). In many cases, docosadienoate and docosahexaenoic acids were present in higher amounts at the first three ripening stages, and at stages IV and V, their contents decreased. However, there is no research on the fatty acids such as eicosadienoate, eicosatrienoate, arachidonic, eicosapentaenoic, docosadienoate, and docosahexaenoic acids of rosehip seeds at different ripening stages.

#### 2.2.2. Monounsaturated Fatty Acids

The total amount of MUFAs was found to vary from 14.67% in *Rosa rugosa* ‘Alba’ seeds at ripening stage I to 18.89% in *Rosa canina* seeds at ripening stage II of the total fatty acids (Table 2). The amounts of total MUFAs in the seeds of *Rosa canina* were the highest and significantly different from those of the *Rosa rugosa*, *Rosa rugosa* ‘Alba’, and *Rosa rugosa* ‘Rubra’. This study showed a significant increase in the total amount of MUFAs during the ripening period.

The evaluated rosehip seeds contained six monounsaturated fatty acids: oleic, palmitoleic, heptadecanoate, eicosenoate, erucic, and nervonic acids. During all ripening stages, oleic acid was the most abundant (13.50 to 18.16% of the total fatty acids), followed by eicosenoate acid, while the rest of the fatty acids were minor and ranged from 0.030 to 0.340%. Güneş et al. [19] have documented that in all the studied rosehip seeds MUFAs ranged between 13.15% (*Rosa dumalis ssp*. boissieri) and 40.31% (*Rosa dumalis*), with oleic acid being dominant among MUFAs (13.12% to 40.26% of the total fatty acids) in all the seed samples. Çelik et al. [3] reported higher amounts of oleic acid (20.35–23.03%) in the seeds of 5 *Rosa* species (*Rosa canina*, *Rosa pulverulanta*, *Rosa dumalis* subsp. Boissieri, *Rosa iberica* and *Rosa heckeliana* subsp. *Vanheurckiana*) in comparison with our results. Some of the differences among the studies may be due to different *Rosa* species, different environmental and climatic conditions, as well as different oil extraction methods [21].

The oleic acid was also found to increase during all ripening stages (Table 2). Significantly, the highest content of this fatty acid (18.16%) was determined in *Rosa canina* seeds at ripening stage V. In *Rosa rugosa* and *Rosa rugosa* ‘Rubra’ seeds, oleic acid was significantly higher at stages IV and V than at the other 3 ripening stages. Significantly, the highest amount of this fatty acid in the seeds of *Rosa rugosa* ‘Alba’ was determined at ripening stage IV (14.16%), while the lowest content was determined at stage I (13.50%) compared with the other ripening stages. Güneş et al. [18] have also found that during maturation, the total amount of MUFAs and oleic acid increased.

The amount of eicosenoate acid decreased with advancing maturity, ranging from 0.740% for stage II (*Rosa rugosa* ‘Rubra’ seeds) to 0.315% for stage V (*Rosa canina* seeds) (Table 2). The *Rosa rugosa* and *Rosa rugosa* ‘Rubra’ seeds had significantly the highest amounts of this fatty acid at ripening stage II, but for *Rosa rugosa* ‘Alba’ seeds it was the highest at stages I and II with no significant differences between them. The seeds of *Rosa canina* showed the lowest amounts of eicosenoate acid at all ripening stages compared with the other tested rosehip seeds. Although the amount of this fatty acid in *Rosa canina* seeds did not significantly differ among ripening stages I, II, III, and IV, it was significantly the lowest at ripening stage V.

The palmitoleic acid was found to decrease during ripening as well. The seeds of the *Rosa canina* and *Rosa rugosa* ‘Alba’ had the highest amount of this fatty acid at ripening stage I (0.090% and 0.230%, respectively) and II (0.090% and 0.230%, respectively), with no significant differences between them. The amount of palmitoleic acid in *Rosa rugosa* ‘Rubra’ seeds was significantly the lowest at ripening stage V (0.180%), but it did not significantly differ among ripening stages I, II, III, and IV (0.233, 0.230, 0.225, and 0.213%, respectively). In addition, *Rosa canina* seeds had the lowest amount of palmitoleic acid at all ripening stages. Güney [21] has reported that the amount of palmitoleic acid of *Rosa canina* seeds was 0.05%.

Our results revealed that heptadecanoate was less affected by fruit maturity than the other fatty acids. No significant differences were determined in the amounts of this fatty acid in the studied rosehip seeds at different ripening stages. The amount of erucic acid decreased during ripening in all seed samples. *Rosa canina* and *Rosa rugosa* showed higher amounts of this fatty acid at the ripening stage I than at the other stages. *Rosa rugosa* ‘Rubra’ seeds showed the highest amounts of erucic acid at all ripening stages compared with the other studied seeds. Moreover, the seeds of this cultivar had the highest amount of this fatty acid at ripening stages I and II. In the seeds of *Rosa rugosa* ‘Alba’, the amount of erucic acid also was significantly the highest (0.205%) at ripening stage II.

However, there is very limited published data on the contents of other minor monounsaturated fatty acids (palmitoleic, heptadecanoate, eicosenoate, erucic, and nervonic acids) in rosehip seeds at different ripening stages.

It has been documented that unsaturated (MUFAs + PUFAs) fatty acids are chemically unstable and easily oxidized [25], therefore, a high amount of these fatty acids in rosehip seed oil makes it susceptible to oxidation as well as degradation. In addition, it can be deduced that reduction of fat content in the seed samples during ripening stage V is the result of the breakdown of some unsaturated fatty acids.

### 2.3. Saturated Fatty Acids

The results averaged over the two experimental years suggest that the content of saturated fatty acids (SFAs) in the rosehip seeds depended on the species/cultivar and ripening stage (Table 3). Depending on the ripening stage, the SFAs amount of the *Rosa canina* ranged 5.96–7.36%, *Rosa Rugosa* 5.22–6.35%, *Rosa Rugosa* ‘Alba’ 5.41–6.15%, and of *Rosa Rugosa* ‘Rubra’ 5.40–6.48%. Çelik et al. [3] observed the seed oil amounts as 8.84% for *Rosa dumalis var.* Boissieri, 8.49% for *Rosa pulverulenta*, 8.50% *Rosa canina* L., 7.77% for *Rosa iberica*, and 7.39% for *Rosa heckeliana* subsp. *Vanheurckiana*. Our research showed that the seeds of *Rosa canina* harvested at ripening stage V had significantly the highest amounts of total SFAs (7.36%). The seeds of *Rosa canina*, *Rosa Rugosa* ‘Alba’, and *Rosa Rugosa* ‘Rubra’ harvested at the ripening stage I had the highest contents of total SFAs, 6.35%, 6.15%, and 6.48%, respectively. Güneş et al. [19] reported that harvest time had an effect on the content of saturated fatty acids. In the seeds of *Rosa canina* and *Rosa villosa*, it ranged between 7.47–8.45 and 6.12–7.31%, respectively.

In our study, thirteen saturated fatty acids were identified in the rosehip seeds: caproic, lauric, myristic, pentadecanoate, palmitic, heptadecanoate, stearic, nonadecanoate, arachidic, heneicosylic, behenic, tricosanoate, and lignoceric acids (Table 3). Koç [26] has identified seven fatty acids in the seeds of five rosehip genotypes and found a great variation among the genotypes.

Our data showed that the dominant fatty acid was palmitic acid, whose content ranged from 2.730 to 3.650% (Table 3). Murathan et al. [27] investigated four rosehip species, *Rosa canina*, *Rosa pimpinellifolia*, *Rosa dumalis* and *Rosa villosa*, and found that palmitic acid (5.50–8.27%) was the major fatty acid in the seeds. Other scientists have also reported that the main acid in rosehip seeds was palmitic acid (3.10–10.13%) [5,28,29]. In our study, significantly, the highest content of this acid was identified in the seed samples of *Rosa rugosa*, *Rosa rugosa* ‘Alba’, and *Rosa rugosa* ‘Rubra’ harvested at ripening stage I, i.e., it was by on average 16.97%, 16.84%, and 15.03%, respectively, compared with the other ripening stages. The seeds of *Rosa rugosa* harvested at stage V contained a significantly (8.06%) higher content of this fatty acid compared with the other ripening stages.

The second dominant saturated fatty acid in rosehip seeds was found to be stearic acid (Table 3), which agreed with the findings published by Barros et al. [25]. Our results show that the stearic acid content in the samples ranged from 0.975% to 2.76%. These data are in line with those obtained in previous studies on rosehip seeds by Fromm et al. [29] and Ilyasoğlu [5]. Significantly, the highest stearic acid contents in the seeds of *Rosa canina* and *Rosa rugosa* were determined at ripening stage V (2.76 and 1.07%, respectively), while in the seeds of *Rosa canina* at stages III, IV, and V, and in the seeds of *Rosa rugosa* at ripening stage IV. Barros et al. [28] established the highest amount of stearic acid (4.64%) in fully ripe rosehip seeds. It has been suggested that harvest time exerts an influence on the fatty acids content of some rosehips [19].

In the seeds of *Rosa Rugosa*, *Rosa rugosa* ‘Alba’, and *Rosa rugosa* ‘Rubra’, significantly, the highest amounts of arachidic acid were determined at ripening stages I and II, while in the seeds of *Rosa canina* at stage V. The study of Çelik et al. [3] on fatty acid contents in the seeds of five rosehip species grown in Hakkâri has found arachidic acid content in these seeds to range between 0.94–1.29% Ilyasoğlu [5] has reported a similar finding (1.00%).

Neither the rosehip species/cultivar nor ripening stage had a significant impact on the contents of lauric, myristic, heptadecanoate, nonadecanoate, and heneicosylic acids in the seeds (Table 3). The study by Kumari et al. [30] has documented that heptadecanoate content of Cleome viscosa accessions from Faridabad, Hyderabad, Surajkund, Jaipur, and Delhi in India varied from 0.20% to 0.70%. In all our investigated seed samples, the contents of lauric and myristic acids ranged between 0.013–0.030% and 0.035–0.048%, respectively. However, a higher content (3.58–4.80%) of lauric acid was reported by Ercisli et al. [31]. Dobreva et al. [32] investigated rosehip seeds; however, the amount of myristic acid was found to be below the detection level.

Our results indicate that the behenic acid content in all rosehip samples ranged from 0.190 to 0.385% (Table 3). In contrast, Güneş et al. [19] found lower contents (0.00–0.07%) of behenic acid in the seeds of five rosehip species harvested six times per season. In our study, the highest contents of this acid were determined in the seeds of *Rosa canina*, *Rosa rugosa*, and *Rosa rugosa* ‘Rubra’ harvested at ripening stage I (0.32, 0.38, and 0.38%, respectively), while in the seeds of *Rosa rugosa* ‘Alba’ (0.39%) at stage V.

The effect of rosehip ripening stage and species/cultivar on the amount of lignoceric acid in the seeds was highly variable. The contents of lignoceric acid in all investigated seeds harvested five times per growing season were found to range between 0.105% and 1.005%. Significantly, the highest amount of lignoceric acid was detected in the seeds of *Rosa rugosa* at ripening stage V when the fruit pulp is mostly softened. Güneş et al. [19] suggested that harvest time had no significant effect on the lignoceric acid amount in the rosehip seeds of *Rosa canina* and varied from 0.01 to 0.02%.

At the end of the experiment, at ripening stages IV and V, the seeds of *Rosa canina* and *Rosa rugosa* contained the highest amounts of tricosanoate acid and there were no significant differences between the samples. The seeds of *Rosa rugosa* ‘Alba’ and *Rosa rugosa* ‘Rubra’ contained the highest amounts of this acid at ripening stages III, IV, and V.

Our study showed that ripening stage had no significant effect on the accumulation of pentadecanoate acid in the seeds of *Rosa canina* and *Rosa rugosa* ‘Alba’. However, in the seeds of *Rosa rugosa*, the highest amounts of this acid were determined at stage I and in the seeds of *Rosa rugosa* ‘Rubra’ at stages I, II, and III.

The *Rosa rugosa* ‘Rubra’ seeds harvested at ripening stages I, II, III, and IV had significantly the highest amounts of caproic acid compared with the other rosehip seeds. *Rosa canina* and *Rosa rugosa* reached the highest amounts of this acid at ripening stage I, while *Rosa rugosa* ‘Alba’ at stage II.

Saturated fatty acids such as heneicosylic, heptadecanoate, nonadecanoate, tricosanoate, pentadecanoate, and caproic acids were identified and quantified in the rosehip seeds in our study. However, there is a lack of published research on these fatty acids in *Rosa* spp. as well as the influence of the ripening stage on their contents.

According to the other researchers, the meteorological conditions and soil fertility may affect the accumulation of fatty acid in seeds of different oilseed crops [33,34,35]; nevertheless, genotypes behave differently under different climatic situations [36]. However, our study showed that the ripening stage and species/genotype differences had a higher effect on the amount of unsaturated fatty acids in tested seed samples than the climatic conditions. It could because the meteorological conditions in 2018 and 2019 were similar. Both years were warmer and had drier weather compared to the standard climate normal (30-year average from 1981 to 2010). Berti et al. [34] reported that nitrogen, phosphorus, and potassium rates had no effect on the composition of the seed oil. Seed oil content and oil composition were also not affected by phosphorus and potassium nutrients. From our experiment, all rosehip plants were grown under the same conditions in the same field with similar soil properties; therefore, it cannot be argued that soil had an influence on compositions of fatty acids.

## 3. Materials and Methods

### 3.1. Field Experiment

**Figure 2:**
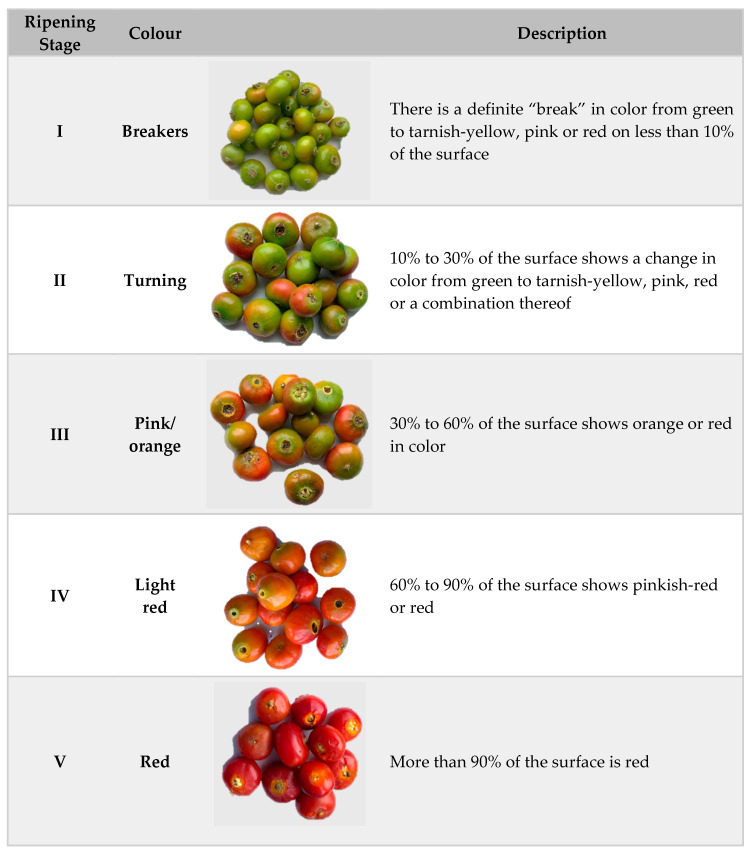
Fruit ripening stages of *Rosa* samples (photos by B. Medveckienė).

A two-factor field experiment with two rosehip species and two cultivars, *Rosa rugosa*, *Rosa canina*, *Rosa rugosa* cv. ‘Rubra’ and *Rosa rugosa* cv. ‘Alba’, was conducted during the two growing seasons (2018 and 2019) on an organically managed farm (certificate No. SER-K-17-01478) in Pakruojis District, Lithuania (56°10′29.0″ N 23°49′02.6″ E). The fruits were harvested five times per season at five ripening stages: stage I—when the fruit color changed slightly from green to yellow, stage II—when the fruit color changed to yellow or red, stage III—when the fruit color changed to light orange or red, stage IV—when the fruit became pinkish-red or red depending on the species, stage V—when the fruit color was red, pulp mostly softened (Figure 2).

The experimental plots were arranged in a randomized design with four replicates. The total experimental area was 2000 m^2^. The distance between the rows was 4 m, and the distance between the rosehip shrubs was 2 m. The shrubs were planted in 2011.

The weather conditions during the rosehip vegetation period in 2018–2019 are shown in Table 4.

The year 2018 and 2019 were warmer by 2.5 and 1.5 °C, respectively, compared with the standard climate normal (SCN). During the vegetation period of 2018 and 2019, there was a drier climate, on average 86.4 and 148.9 mm, respectively, compared to the SCN. Compared with the SCN, the sunshine in both years during the rosehip vegetation period was higher on average at 264 h.

### 3.2. Preparation of Rosehip Seed Samples

Rosehips were hand-picked (1 kg per each species and variety) from four plants from each replication from June to September. Before analyses, the rosehip fruits were cut in half and the seeds were separated. The seeds were frozen at −35 °C and then lyophilized for 24 h using Freeze—Drying Plant Sublimator 3 × 4 × 5 (ZIRBUS Technology GmbH, Bad Grund, Germany). The lyophilized seeds were milled (Grindomix GM 200, Retsch GmbH, Haan, Germany) and stored in sealed containers at 5 °C in the dark until further analysis.

### 3.3. Soil Agrochemical Analyses

Agrochemical analyses of the experimental soil were conducted at the Laboratory of Food Raw Materials, Zootechnical and Agronomic Analyses of Vytautas Magnus University Agriculture Academy. The soil samples were air-dried in open plastic boxes. After removing small stones, root residues and other organic plant parts the samples were crushed. Homogenized soil was sieved through a 1 mm mesh size sieve. The soil samples were analyzed for pH_KCl_, contents of available phosphorus, available potassium, and total nitrogen. Soil pH_KCl_ was measured by the potentiometric method, using a pH-meter in 1NKCl extract [37]. Available phosphorus and potassium were extracted with ammonium-lactate according to the Egner–Riehm–Domingo method [38]. The total nitrogen concentration (mg kg^−1^) was determined by the Kjeldahl method.

The soil of the experimental field was characterized by close to neutral acidity (pH_KCl_ = 6.13–6.82), medium potassium status (K_2_O = 94.8–155.2 mg kg^−1^), and medium phosphorus status (P_2_O_5_ = 121.9–141.2 mg kg^−1^), and total nitrogen content of 2.69%.

### 3.4. Determination of Oil Content of Rosehip Seeds

The oil content of rosehip seeds was measured by the AOAC (Association of Official Analytical Chemists) method no. 920.85 [39], and fat was extracted with n-hexane (60 °C) for 6 h using an automatic Soxhlet apparatus (Gerhardt, Analytical Systems, Germany) following the manufacturer’s guidelines. The rosehip seeds were ground and packed in cellulose extraction thimble and the oil was extracted with petroleum ether (boiling point: 60–90 °C) for 1.5 h. After extraction, the oil was dried at 105 °C for 5 h to remove residual water and petroleum ether. The oil content of the seed samples was calculated on a dry weight basis.

### 3.5. Determination of Fatty Acids Content of Rosehip Seeds

The fatty acid composition of rosehip seeds was determined in accordance with the ISO 5509:2000 standard by using a Clarus 500 (Perkin Elmer) Gas Chromatograph with Split/Splitless Injector Liners. The test sample was dissolved in t-butyl methyl ether, and the methyl esters were prepared by trans-esterification with trimethylsulfonium hydroxide (TMSH). The conditions for this analysis were: capillary column ZEBRON ZB-FAME (30 × 0.2 mm × 0.25 μm), the initial oven temperature was set at 100 °C and held for 2 min. Then, the temperature was increased at the rate 3 °C min^−1^ to 240 °C and held at 240 °C for 5 min. Nitrogen was used as a carrier gas. The injector temperature was 285 °C. Fatty acids were identified by comparing their retention times with those of the standards. Each measurement was performed in triplicate.

### 3.6. Statistical Analysis

The data of the fatty acids content of rosehip seeds were processed by the Microsoft^®^ Excel^®^ 2016 MSO software and the STATISTICA 10 (StatSoft, Inc., Tulsa, OK, USA) package. In this study, two-year averages were submitted, and there were no significant interactions between the year of investigation and treatments (at *p* < 0.05). However, there were significant interactions among rosehip seed samples and ripening stages. The reliability of the results was evaluated by a two-way analysis of variance, using the ANOVA software package. The statistical significance of differences between the means was estimated by Fisher’s LSD test (*p* < 0.05).

## 4. Conclusions

The current study showed that the content and composition of fatty acids in rosehip seeds varied considerably between rosehip species/cultivars and ripening stages. The ripening stage had a significant effect on the amount of fat in seeds. In all investigated seed samples, significantly, the highest contents of fat were established at ripening stage IV. Among the saturated fatty acids, palmitic and stearic acids were the most prevalent. The amount of monounsaturated fatty acids, significantly, increased during the ripening period. Among the monounsaturated fatty acids, oleic acid was recorded as the major fatty acid in the seeds. Total polyunsaturated fatty acids accounted for from 73.88% to 79.52% of the fatty acid profile, with a predominance of fatty acids in seeds: linoleic acid and linolenic acid. Results from this investigation have revealed that using seed oil from the early stages of fruit maturity may improve the quality of both the seed oil (feedstock) and hence of derived biodiesel in contrast to the final maturity stage.

The results of this study will be useful to choose suitable species/cultivar of rosehip for desirable fatty acids and optimal harvest time of seeds. It can be concluded that the rosehip seeds, especially seeds of *Rosa rugosa* ‘Alba’ at ripening stage IV, could be an important source of polyunsaturated fatty acids.

The findings of the present study suggest that rosehip seeds, the by-product of the rosehip industry, could be an important source of polyunsaturated fatty acids.

## Figures and Tables

**Table 1:** Amount of polyunsaturated fatty acids in the organic rosehip (*Rosa* spp.) seeds (%).

Rosehip Seed Samples	Ripening Stages				
	I	II	III	IV	V
Linoleic acid (C18:2)					
*Rosa canina*	50.63 ^e,f^	50.24 ^d,e^	50.22 ^d^	51.12 ^f^	54.28 ^h^
*Rosa rugosa*	48.69 ^b,c^	47.68 ^a,b^	48.68 ^b,c^	48.71 ^c^	48.26 ^b^
*Rosa rugosa* ‘Alba’	49.76 ^c,d^	50.58 ^e^	51.62 ^f,g^	50.24 ^d,e^	49.76 ^c,d^
*Rosa rugosa* ‘Rubra’	48.61 ^b^	48.46 ^b^	48.51 ^b^	48.64 ^b,c^	47.38 ^a^
Linolenic acid (C18:3)					
*Rosa canina*	24.31 ^b,c^	25.20 ^d^	24.33 ^c^	24.26 ^b^	19.31 ^a^
*Rosa rugosa*	29.70 ^k^	30.64 ^l^	29.57 ^j,k^	29.37 ^y,j^	29.43 ^j^
*Rosa rugosa* ‘Alba’	28.88 ^h,i^	27.85 ^e,f^	27.34 ^e^	28.49 ^g,h^	29.26 ^y^
*Rosa rugosa* ‘Rubra’	29.09 ^i^	28.27 ^f,g^	29.13 ^i,y^	28.70 ^h^	30.64 ^l^
Eicosadienoic acid (C20:2)					
*Rosa canina*	0.110 ^a,b^	0.113 ^b^	0.110 ^a,b^	0.103 ^a,b^	0.100 ^a^
*Rosa rugosa*	0.183 ^e^	0.180 ^e^	0.173 ^d,e^	0.163 ^c,d^	0.170 ^c,d^
*Rosa rugosa* ‘Alba’	0.180 ^e^	0.180 ^e^	0.173 ^d,e^	0.180 ^e^	0.180 ^e^
*Rosa rugosa* ‘Rubra’	0.183 ^e^	0.183 ^e^	0.173 ^d,e^	0.160 ^c^	0.163 ^c,d^
Eicosatrienoic acid (C20:3)					
*Rosa canina*	0.130 ^h,i^	0.123 ^g,h^	0.125 ^h^	0.140 ^i^	0.045 ^a^
*Rosa rugosa*	0.131 ^h,i^	0.111 ^e,f^	0.101 ^c,d^	0.091 ^b,c^	0.091 ^b,c^
*Rosa rugosa* ‘Alba’	0.121 ^g,h^	0.123 ^g,h^	0.130 ^h,i^	0.113 ^f,g^	0.120 ^g,h^
*Rosa rugosa* ‘Rubra’	0.130 ^h,i^	0.113 ^f,g^	0.103 ^d,e^	0.090 ^b,c^	0.080 ^b^
Arachidonic acid (C20:4)					
*Rosa canina*	0.110 ^c^	0.081 ^b^	0.082 ^b^	0.060 ^a^	0.061 ^a^
*Rosa rugosa*	0.071 ^a,b^	0.070 ^a,b^	0.071 ^a,b^	0.071 ^a,b^	0.060 ^a^
*Rosa rugosa* ‘Alba’	0.080 ^b^	0.081 ^b^	0.071 ^a,b^	0.071 ^a,b^	0.071 ^a,b^
*Rosa rugosa* ‘Rubra’	0.123 ^c^	0.133 ^d^	0.115 ^c^	0.115 ^c^	0.080 ^b^
Eicosapentaenoic acid (C20:5)					
*Rosa canina*	0.01 ^b^	0.000 ^a^	0.000 ^a^	0.000 ^a^	0.020 ^c^
*Rosa rugosa*	0.000 ^a^	0.000 ^a^	0.000 ^a^	0.000 ^a^	0.000 ^a^
*Rosa rugosa* ‘Alba’	0.020 ^c^	0.000 ^a^	0.000 ^a^	0.001 ^b^	0.000 ^a^
*Rosa rugosa* ‘Rubra’	0.050 ^e^	0.050 ^e^	0.040 ^d^	0.060 ^f^	0.020 ^c^
Docosadienoic acid (C22:2)					
*Rosa canina*	0.010 ^a^	0.093 ^e^	0.082 ^c^	0.071 ^c^	0.060 ^b^
*Rosa rugosa*	0.020 ^a^	0.090 ^d,e^	0.091 ^d,e^	0.081 ^c^	0.081 ^c^
*Rosa rugosa* ‘Alba’	0.111 ^f^	0.105 ^f^	0.021 ^a^	0.105 ^f^	0.091 ^d,e^
*Rosa rugosa* ‘Rubra’	0.192 ^i^	0.192 ^i^	0.171 ^h^	0.141 ^g^	0.111 ^f^
Docosahexaenoic acid (C22:6)					
*Rosa canina*	0.021 ^b,c^	0.051 ^h,i^	0.052 ^i^	0.041 ^e,f,g^	0.030 ^c,d,e^
*Rosa rugosa*	0.030 ^c,d,e^	0.023 ^c,d^	0.031 ^c,d,e^	0.030 ^c,de^	0.000 ^a^
*Rosa rugosa* ‘Alba’	0.040 ^e,f,g^	0.010 ^a,b^	0.041 ^e,f,g^	0.031 ^c,d^	0.041 ^e,f,g^
*Rosa rugosa* ‘Rubra’	0.040 ^e,f,g^	0.051 ^h,i^	0.035 ^e,f,g^	0.045 ^g,h^	0.033 ^d,e,f^
Sum of PUFAs					
*Rosa canina*	75.31 ^b^	75.91 ^b,c^	75.02 ^b^	75.79 ^b,e^	73.88 ^a^
*Rosa rugosa*	78.82 ^h^	78.81 ^h^	78.72 ^h^	78.51 ^h,g^	78.10 ^e,f^
*Rosa rugosa* ‘Alba’	79.18 ^l^	78.92 ^j^	79.38 ^l^	79.24 ^l^	79.52 ^k^
*Rosa rugosa* ‘Rubra’	78.40 ^g^	77.44 ^d^	78.26 ^f^	77.96 ^de^	78.51 ^h,g^

Note: Different letters in the same column and line represent significant differences between rosehip seed samples and ripening stages, respectively (*p* < 0.05).

**Table 2:** Amount of monounsaturated fatty acids in the organic rosehip (*Rosa* spp.) seeds (%).

Rosehip Seed Samples	Ripening Stages				
	I	II	III	IV	V
Palmitoleic acid (C16:1)					
*Rosa canina*	0.090 ^a^	0.090 ^a^	0.081 ^b^	0.060 ^c^	0.070 ^b,c^
*Rosa rugosa*	0.233 ^f^	0.213 ^e,f^	0.180 ^d^	0.183 ^d^	0.233 ^f^
*Rosa rugosa* ‘Alba’	0.230 ^f^	0.230 ^f^	0.190 ^d,e^	0.203 ^d,e^	0.193 ^d,e^
*Rosa rugosa* ‘Rubra’	0.233 ^f^	0.230 ^f^	0.225 ^e,f^	0.213 ^e,f^	0.180 ^d^
Heptadecanoic acid (C17:1)					
*Rosa canina*	0.040 ^a^	0.040 ^a^	0.040 ^a^	0.040 ^a^	0.040 ^a^
*Rosa rugosa*	0.053 ^a,b,c^	0.053 ^a,b,c^	0.063 ^c^	0.063 ^c^	0.063 ^c^
*Rosa rugosa* ‘Alba’	0.043 ^a,b^	0.053 ^a,b,c^	0.063 ^c^	0.063 ^c^	0.063 ^c^
*Rosa rugosa* ‘Rubra’	0.055 ^b,c^	0.055 ^b,c^	0.060 ^c^	0.058 ^c^	0.058 ^c^
Oleic acid (C18:1)					
*Rosa canina*	17.24 ^f^	17.08 ^g^	17.18 ^f^	17.18 ^f^	18.16 ^h^
*Rosa rugosa*	13.52 ^a^	14.05 ^c^	14.68 ^c^	15.12 ^e^	15.29 ^e^
*Rosa rugosa* ‘Alba’	13.50 ^a^	13.68 ^b^	13.85 ^b^	14.16 ^c^	13.81 ^b^
*Rosa rugosa* ‘Rubra’	13.70 ^b^	14.78 ^c^	14.48 ^c^	15.16 ^e^	14.98 ^d^
Eicosenoic acid (C20:1)					
*Rosa canina*	0.452 ^b^	0.455 ^b^	0.450 ^b^	0.440 ^b^	0.315 ^a^
*Rosa rugosa*	0.680 ^i^	0.712 ^y^	0.650 ^g^	0.630 ^f^	0.585 ^c^
*Rosa rugosa* ‘Alba’	0.660 ^h,i^	0.652 ^h^	0.592 ^c,d^	0.580 ^c^	0.605 ^d,e^
*Rosa rugosa* ‘Rubra’	0.705 ^y^	0.740 ^j^	0.675 ^i^	0.625 ^e^	0.580 ^c^
Erucic acid (C22:1)					
*Rosa canina*	0.230 ^f^	0.195 ^e^	0.170 ^c,d^	0.143 ^a^	0.160 ^b,c^
*Rosa rugosa*	0.180 ^d^	0.135 ^a^	0.160 ^bc^	0.154 ^a,b^	0.120 ^a^
*Rosa rugosa* ‘Alba’	0.180 ^d^	0.205 ^f^	0.173 ^c,d^	0.183 ^d^	0.153 ^a,b^
*Rosa rugosa* ‘Rubra’	0.323 ^h^	0.340 ^h^	0.295 ^g^	0.295 ^g^	0.170 ^c,d^
Nervonic acid (C24:1)					
*Rosa canina*	0.255 ^f,g^	0.265 ^f,g^	0.250 ^f,g^	0.288 ^g^	0.030 ^a^
*Rosa rugosa*	0.170 ^de^	0.153 ^d^	0.125 ^c^	0.125 ^c^	0.180 ^e^
*Rosa rugosa* ‘Alba’	0.060 ^b^	0.175 ^e^	0.175 ^e^	0.175 ^e^	0.240 ^f^
*Rosa rugosa* ‘Rubra’	0.115 ^c^	0.125 ^c^	0.115 ^c^	0.080 ^b^	0.125 ^c^
Sum of MUFAs					
*Rosa canina*	18.39 ^j^	18.13 ^i^	18.89 ^k^	18.15 ^i^	18.76 ^k^
*Rosa rugosa*	14.83 ^b^	15.30 ^d^	15.85 ^e^	16.27 ^g^	16.47 ^h^
*Rosa rugosa* ‘Alba’	14.67 ^a^	14.96 ^b,c^	15.04 ^b,c^	15.35 ^d^	15.05 ^b,c^
*Rosa rugosa* ‘Rubra’	15.14 ^c^	16.27 ^g^	15.80 ^e^	16.43 ^h^	16.09 ^f^

Note: Different letters in the same column and line represent significant differences between rosehip seed samples and ripening stages, respectively (*p* < 0.05).

**Table 3:** Amount of saturated fatty acids in the organic rosehip (*Rosa* spp.) seeds (%).

Rosehip Seed Samples	Ripening Stages				
	I	II	III	IV	V
Caproic acid (C:6)					
*Rosa canina*	0.040 ^d^	0.020 ^b^	0.030 ^c^	0.025 ^c^	0.000 ^a^
*Rosa rugosa*	0.020 ^b,c^	0.005 ^a,b^	0.015 ^b,c^	0.000 ^a^	0.020 ^b^
*Rosa rugosa* ‘Alba’	0.020 ^b^	0.025 ^c^	0.000 ^a^	0.000 ^a^	0.020 ^b^
*Rosa rugosa* ‘Rubra’	0.070 ^e^	0.070 ^e^	0.065 ^e^	0.065 ^e^	0.040 ^d^
Lauric acid (C:12)					
*Rosa canina*	0.020 ^a,b^	0.020 ^a,b^	0.020 ^a,b^	0.015 ^a^	0.015 ^a^
*Rosa rugosa*	0.020 ^a,b^	0.020 ^a,b^	0.015 ^a^	0.015 ^a^	0.020 ^a,b^
*Rosa rugosa* ‘Alba’	0.030 ^b^	0.013 ^a^	0.013 ^a^	0.010 ^a^	0.020 ^a,b^
*Rosa rugosa* ‘Rubra’	0.010 ^a^	0.010 ^a^	0.020 ^a,b^	0.020 ^a,b^	0.015 ^a^
Myristic acid (C:14)					
*Rosa canina*	0.040 ^a^	0.040 ^a^	0.040 ^a^	0.035 ^a^	0.035 ^a^
*Rosa rugosa*	0.045 ^a^	0.045 ^a^	0.040 ^a^	0.040 ^a^	0.040 ^a^
*Rosa rugosa* ‘Alba’	0.048 ^a^	0.048 ^a^	0.040 ^a^	0.040 ^a^	0.040 ^a^
*Rosa rugosa* ‘Rubra’	0.040 ^a^	0.040 ^a^	0.040 ^a^	0.030 ^a^	0.045 ^a^
Pentadecanoic acid (C:15)					
*Rosa canina*	0.030 ^a^	0.030 ^a^	0.030 ^a^	0.030 ^a^	0.030 ^a^
*Rosa rugosa*	0.050 ^d^	0.040 ^b,c^	0.030 ^a^	0.030 ^a^	0.033 ^a,b^
*Rosa rugosa* ‘Alba’	0.041 ^b,c,d^	0.041 ^b,c,d^	0.040 ^b,c^	0.041 ^b,c,d^	0.041 ^b,c,d^
*Rosa rugosa* ‘Rubra’	0.050 ^d^	0.045 ^c,d^	0.041 ^b,c,d^	0.035 ^a,b^	0.030 ^a^
Palmitic acid (C:16)					
*Rosa canina*	2.86 ^d^	2.78 ^a,b^	2.76 ^a,b^	2.73 ^a^	3.025 ^f^
*Rosa rugosa*	3.62 ^j^	3.32 ^h^	2.98 ^f^	2.88 ^d,e^	2.84 ^c,d^
*Rosa rugosa* ‘Alba’	3.62 ^j^	3.40 ^i^	2.98 ^f^	2.88 ^d,e^	2.80 ^b,c^
*Rosa rugosa* ‘Rubra’	3.65 ^j^	3.46 ^y^	3.18 ^g^	2.92 ^e^	2.84 ^c,d^
Heptadecanoic acid (C:17)					
*Rosa canina*	0.060 ^a^	0.060 ^a^	0.060 ^a^	0.060 ^a^	0.070 ^a^
*Rosa rugosa*	0.060 ^a^	0.050 ^a^	0.055 ^a^	0.050 ^a^	0.050 ^a^
*Rosa rugosa* ‘Alba’	0.060 ^a^	0.060 ^a^	0.053 ^a^	0.050 ^a^	0.050 ^a^
*Rosa rugosa* ‘Rubra’	0.060 ^a^	0.055 ^a^	0.055 ^a^	0.050 ^a^	0.060 ^a^
Stearic acid (C:18)					
*Rosa canina*	1.76 ^h,i^	1.73 ^h^	1.76 ^h,i^	1.790 ^i^	2.76 ^y^
*Rosa rugosa*	1.06 ^c,d,e^	1.02 ^a,b,c^	1.04 ^b,c,d^	1.01 ^a,b^	1.07 ^d,e^
*Rosa rugosa* ‘Alba’	0.975 ^a^	1.08 ^d,e^	1.13 ^f,g^	1.08 ^e,f^	1.08 ^e,f^
*Rosa rugosa* ‘Rubra’	1.08 ^d,e^	1.06 ^b,c,d,e^	1.08 ^d,e^	1.14 ^g^	1.14 ^g^
Nonadecanoic acid (C:19)					
*Rosa canina*	0.050 ^a,b,c^	0.050 ^a,b,c^	0.045 ^a,b^	0.055 ^a,b,c^	0.050 ^a,b,c^
*Rosa rugosa*	0.050 ^a,b,c^	0.055 ^a,b,c^	0.060 ^b,c^	0.055 ^a,b,c^	0.060 ^b,c^
*Rosa rugosa* ‘Alba’	0.060 ^b,c^	0.041 ^a,b^	0.050 ^a,b,c^	0.050 ^a,b,c^	0.061 ^b,c^
*Rosa rugosa* ‘Rubra’	0.045 ^a,b^	0.060 ^b,c^	0.060 ^b,c^	0.066 ^b,c^	0.040 ^a,b^
Arachidic acid (C:20)					
*Rosa canina*	0.815 ^i,y^	0.735 ^b,c,d,e^	0.800 ^g,h,i,y^	0.770 ^e,f,g^	1.010 ^j^
*Rosa rugosa*	0.790 ^g,h,i^	0.785 ^g,h,i^	0.705 ^a,b,c^	0.690 ^a^	0.700 ^a,b^
*Rosa rugosa* ‘Alba’	0.810 ^h,i,y^	0.775 ^f,g,h^	0.745 ^d,e,f^	0.742 ^c,d,e^	0.730 ^b,c,d^
*Rosa rugosa* ‘Rubra’	0.830 ^y^	0.835 ^y^	0.790 ^g,h,i^	0.765 ^d,e,f,g^	0.735 ^b,c,d,e^
Heneicosylic acid (C:21)					
*Rosa canina*	0.030 ^a^	0.030 ^a^	0.030 ^a^	0.030 ^a^	0.030 ^a^
*Rosa rugosa*	0.045 ^b,c^	0.040 ^a,b,c^	0.040 ^a,b,c^	0.040 ^a,b,c^	0.040 ^a,b,c^
*Rosa rugosa* ‘Alba’	0.040 ^a,b,c^	0.040 ^a,b,c^	0.040 ^a,b,c^	0.040 ^a,b,c^	0.040 ^a,b,c^
*Rosa rugosa* ‘Rubra’	0.050 ^c^	0.035 ^a,b^	0.035 ^a,b^	0.035 ^a,b^	0.040 ^a,b,c^
Behenic acid (C:22)					
*Rosa canina*	0.320 ^d^	0.315 ^b,c,d^	0.315 ^b,c,d^	0.310 ^b,c^	0.190 ^a^
*Rosa rugosa*	0.375 ^g,h^	0.345 ^e,f^	0.315 ^b,c,d^	0.310 ^b,c^	0.315 ^b,c,d^
*Rosa rugosa* ‘Alba’	0.310 ^b,c^	0.345 ^e,f^	0.345 ^e,f^	0.330 ^d,e^	0.385 ^h^
*Rosa rugosa* ‘Rubra’	0.375 ^g,h^	0.360 ^f,g^	0.335 ^d,e^	0.310 ^b,c^	0.300 ^b^
Tricosanoic acid (C:23)					
*Rosa canina*	0.020 ^a^	0.020 ^a^	0.035 ^b,c^	0.040 ^b,c,d,e^	0.040 ^b,c,d,e^
*Rosa rugosa*	0.030 ^a,b^	0.020 ^a^	0.018 ^a^	0.038 ^b,c,d^	0.038 ^b,c,d^
*Rosa rugosa* ‘Alba’	0.020 ^a^	0.061 ^g^	0.050 ^d,e,f,g^	0.051 ^e,f,g^	0.051 ^e,f,g^
*Rosa rugosa* ‘Rubra’	0.020 ^a^	0.020 ^a^	0.055 ^f,g^	0.045 ^c,d,e,f^	0.050 ^d,e,f,g^
Lignoceric acid (C:24)					
*Rosa canina*	0.245 ^g^	0.130 ^b,c,d^	0.135 ^c,d^	0.195 ^f^	0.130 ^b,c,d^
*Rosa rugosa*	0.165 ^e^	0.165 ^e^	0.110 ^a,b^	0.105 ^a^	0.170 ^e^
*Rosa rugosa* ‘Alba’	0.130 ^b,c,d^	0.205 ^f^	0.120 ^a,b,c,d^	0.115 ^a,b,c^	0.120 ^a,b,c,d^
*Rosa rugosa* ‘Rubra’	0.210 ^f^	0.245 ^g^	0.195 ^f^	0.140 ^d^	1.005 ^h^
Sum of SFAs					
*Rosa canina*	6.30 ^g^	5.96 ^c,d^	6.09 ^d,e^	6.06 ^d^	7.36 ^i^
*Rosa rugosa*	6.35 ^g^	5.89 ^c,d^	5.43 ^a,b^	5.22 ^a^	5.43 ^a,b^
*Rosa rugosa* ‘Alba’	6.15 ^f^	6.12 ^e^	5.58 ^b,c^	5.41 ^a,b^	5.43 ^a,b^
*Rosa rugosa* ‘Rubra’	6.48 ^h^	6.29 ^g^	5.94 ^c,d^	5.61 ^b,c^	5.40 ^a,b^

Note: Different letters in the same column and line represent significant differences between rosehip seed samples and ripening stages, respectively (*p* < 0.05).

**Table 4:** Weather conditions during the rosehip vegetation period in 2018–2019 (Šiauliai meteorological station, Lithuania).

Years	Months						
	April	May	June	July	August	September	Average
Air temperature, °C							
2018	10.2	17.1	17.4	19.6	19.2	14.5	16.3
2019	9.1	13.4	21.2	17.2	18.2	12.5	15.3
SCN *	7.0	12.8	15.7	18.0	17.1	12.0	13.8
Rainfall, mm							Sum
2018	42.6	27.5	16.0	107.9	65.6	57.0	316.6
2019	0.7	28.6	27.5	50.3	100.5	46.5	254.1
SCN	43	57	73	89	75	66	403
Sunshine, h							Sum
2018	248	365	286	210	276	207	1592
2019	329	232	349	233	264	189	1596
SCN	179	252	246	260	237	154	1328

* SCN—Standard climate normal is the 30-year average from 1981 to 2010.
